# Genotyping by Sequencing for SNP-Based Linkage Map Construction and QTL Analysis of Chilling Requirement and Bloom Date in Peach [*Prunus persica* (L.) Batsch]

**DOI:** 10.1371/journal.pone.0139406

**Published:** 2015-10-02

**Authors:** Douglas Gary Bielenberg, Bradley Rauh, Shenghua Fan, Ksenija Gasic, Albert Glenn Abbott, Gregory Lynn Reighard, William R. Okie, Christina Elizabeth Wells

**Affiliations:** 1 Department of Biological Sciences, College of Agriculture, Forestry & Life Sciences, Clemson University, Clemson, South Carolina, 29634–0314, United States of America; 2 Advanced Plant Technology Program, Clemson University, Clemson, South Carolina, 29634, United States of America; 3 Department of Genetics & Biochemistry, College of Agriculture, Forestry & Life Sciences, Clemson University, Clemson, South Carolina, 29634, United States of America; 4 Department of Agricultural and Environmental Sciences, College of Agriculture, Forestry & Life Sciences, Clemson University, Clemson, South Carolina, 29634, United States of America; 5 Southeastern Fruit and Tree Nut Research Laboratory (retired), USDA-ARS, Byron, Georgia, 31008, United States of America; Wuhan Botanical Garden of Chinese Academy of Sciences, CHINA

## Abstract

Low-cost, high throughput genotyping methods are crucial to marker discovery and marker-assisted breeding efforts, but have not been available for many ‘specialty crops’ such as fruit and nut trees. Here we apply the Genotyping-By-Sequencing (GBS) method developed for cereals to the discovery of single nucleotide polymorphisms (SNPs) in a peach F_2_ mapping population. Peach is a genetic and genomic model within the Rosaceae and will provide a template for the use of this method with other members of this family. Our F_2_ mapping population of 57 genotypes segregates for bloom time (BD) and chilling requirement (CR) and we have extensively phenotyped this population. The population derives from a selfed F_1_ progeny of a cross between ‘Hakuho’ (high CR) and ‘UFGold’ (low CR). We were able to successfully employ GBS and the TASSEL GBS pipeline without modification of the original methodology using the *Ape*KI restriction enzyme and multiplexing at an equivalent of 96 samples per Illumina HiSeq 2000 lane. We obtained hundreds of SNP markers which were then used to construct a genetic linkage map and identify quantitative trait loci (QTL) for BD and CR.

## Introduction

High-throughput sequencing platforms such as the Illumina HiSeq® have inspired the development of methods for cost-efficient, genome-wide genotyping of numerous individuals in genetic mapping and population scale studies [1]. These methods achieve cost efficiency through deep multiplexing large numbers of individuals on a sequencing lane and targeting a limited percentage of the genome associated with restriction enzyme loci distributed across all chromosomes. One such method, Genotyping-by-Sequencing (GBS), was developed for low-cost, high-throughput genotyping in maize and has subsequently been used in a number of crop species [2]. GBS is particularly attractive because of the relatively simple bench protocol involved in generating bar-coded samples, which are then combined into a single library for sequencing on the Illumina platform [2]. Our aim was to evaluate the effectiveness with which GBS could be applied to a perennial tree fruit species such as peach [*Prunus persica* (L) Batsch] [3, 4]. Successful application of GBS to peach would suggest that the method is readily applicable to other economically important forest, fruit, and nut species within the Rosaceae.

Chilling requirement (CR) refers to the minimum duration of cold exposure required before dormant buds will bloom in response to bud break-inducing conditions [5]. CR contributes strongly to bloom date (BD), although BD is also affected by an endogenous heat requirement [6]. Growers and breeders must select cultivars whose CR and BD closely match local climatic conditions in order to avoid crop losses due to late frosts or poor bud break due to insufficient chilling [7]. CR and BD therefore impose a constraint on the introduction and spread of new cultivars with superior agronomic performance and marketability.

CR and BD are known to be quantitative genetic traits whose phenotypes vary widely among peach cultivars [8, 9]. Since CR and BD can only be evaluated after individuals reach reproductive maturity (3–4 years for peach), identification of genetic markers linked with CR and BD phenotypes would save time and resources by allowing selection of genotypes at the seedling stage. As a test of the GBS method in *Prunus*, we genotyped a F_2_ peach mapping population segregating for CR and BD for which we have multiple years of phenotypic data. Here we present the successful use of GBS in peach and the identification of multiple QTLs for CR and BD in our F_2_ population.

## Methods

### Plant material

A peach F_2_ population of 57 genotypes was developed at the USDA-ARS Southern Fruit and Tree Nut Research Laboratory (Byron, GA, U.S.A.) by crossing the high chill requirement cultivar Hakuho with the low chill requirement cultivar UFGold and selfing its F_1_ hybrid. The female grandparent ‘Hakuho’ is a white-fleshed commercial peach cultivar originating from Japan that requires approximately 900 chilling hours for spring bloom [9]. The male grandparent ‘UFGold’ is a yellow-fleshed commercial cultivar released by the University of Florida (Gainesville, FL, U.S.A.) breeding program [10] that requires approximately 400 chilling hours for spring bloom.

F_2_ seeds were stratified, germinated and planted in a greenhouse in fall 2003, then transplanted in spring 2004 to Clemson University’s Musser Fruit Research Center (Seneca, SC, U.S.A.). Replicate clones of each tree were produced by rooting current-year stem cuttings during the 2005 growing season and replicate plantings were established on their own roots at the Musser Fruit Research Center in spring 2006. Replicates of each grandparent were also included in these plantings, although the F_1_ tree was no longer available. ‘Hakuho’ was propagated from the original tree used in the cross. Twenty ‘UF Gold’ trees were obtained from a commercial nursery grafted onto ‘Nemaguard’ rootstock.

### Phenotyping

Bloom date was visually scored as the date at which 50% of the floral buds on an individual tree reached full bloom stage. Bloom date of all F_2_ individuals and grandparents was scored in the original 2004 planting each spring from 2006 through 2012. Observations of bloom progression were made every 2–3 days from the onset of floral bud break. Bloom date was recorded as the number of days from January 1^st^ of each year.

Chilling requirement of the F_2_ population and grandparents was determined in two successive winters (2008/2009 and 2009/2010) using the procedures detailed in our previous publication [8]. In brief, temperature-recording data loggers were placed in the canopy of replicate trees and the average temperature was recorded at 10 minute intervals from mid-October through full bloom in late March. Sampling was performed approximately every 100 hours of accumulated chill time below 7.2°C beginning at 200 hours [11].

On each sampling date three branches (>40cm in length with floral buds) were harvested from each of three replicate trees from each genotype. Branch cuttings were brought to a 25°C greenhouse at Clemson University, recut under water, placed in a 1% Floralife solution (Floralife, Inc., Walterboro, SC, U.S.A.) and maintained under a 16 hour photoperiod. Branches were recut and fresh solution supplied after seven days. At 14 d, % floral bud break was scored on all cuttings. A genotype’s CR was considered to be fulfilled when 50% of the flower buds on all cuttings had opened sufficiently for the petals to be visible.

### Genotyping

Genomic DNA for SSR marker screening was isolated as described previously [8]. Genomic DNA for SNP identification was isolated from powdered, freeze-dried leaf tissue of all F_2_ individuals and grandparents using the Dellaporta *et al*. miniprep method [12]. DNA quality was assessed by 260 nm/280 nm absorbance ratios with a Biophotometer 6131 (Eppendorf, Hauppauge, NY). DNA was quantified using the QuantiFluor dsDNA labeling system (Promega, Madison, WI) with a TBS Mini-Fluorometer (Turner Biosystems, Sunnyvale, CA).

A set of 370 simple sequence repeat (SSR) markers from *Prunus* species were screened for polymorphism in the F_2_ mapping population. Markers from all eight major peach linkage groups were selected based on known locations on the T x E *Prunus* reference map [13] and the peach ‘bin map’ [14]. SSR screening was performed as described in Fan *et al*. [8]. SSR origins and references are as described in Fan *et al*. [8].

We followed the ‘Genotyping By Sequencing’ (GBS) method of Elshire *et al*. [2] to generate *Ape*KI-associated DNA fragments for sequencing on the Illumina HiSeq® 2000 platform. Ninety-six double-stranded forward adaptors each with a unique barcode and a single common double-stranded reverse adaptor were created from a set of 194 single-stranded oligonucleotides (IDT, Coralville, IA, U.S.A.). Each adaptor contained a three base overhang for ligation with *Ape*KI digested DNA. The *ApeK*I compatible barcode set was that published in Elshire *et al*. [2].

Adaptors were ligated to restriction-digested DNA from F2 individuals and grandparents following the methods described in [2]. Pooled, amplified libraries were sent to the Cornell University Biotechnology Resource Center for single-end sequencing on the Illumina HiSeq® 2000 platform. Grandparental samples were run in triplicate (three separately barcodes samples), whereas F_2_ individuals were run once. Ninety-six barcoded samples were run on a single Illumina lane, including some samples from a separate experiment. Samples were re-sequenced if less thant 100,000 reads with identifiable barcodes were obtained from the initial run.

Preliminary inspection of the raw fastq files showed low recovery of five expected barcodes. Using Homertools [15] and basic Linux commands, we extracted barcode sequences from all reads and determined the frequency with which each barcode appeared in the sequence file (S1 File). This analysis revealed a systematic sequencing error that produced a single ‘N’ in the seventh position of several of the barcodes. Due to the robust design of the barcode sequences, we were able to unambiguously assign these approximately 1.5 million mis-barcoded reads to their proper samples.

### Processing of sequenced reads

Sequenced reads were processed using default parameters of the TASSEL 3.0 and 4.0 GBS pipeline obtained from the Maize Genetics and Diversity Lab (www.maizegenetics.net) at Cornell University [16]. Assembled scaffolds of the peach genome v1.0 were downloaded from the Genome Database for Rosaceae and used as the reference sequence for alignment of sequenced reads with Bowtie v2.1 [17, 18]. SNP calls for all genotypes were exported as vcf files for filtering with vcftools [19]. SNP calls within a genotype were filtered, retaining only those with a minimum depth of five reads and a genotype quality score of 98 [20]. Filtered vcf files were imported to the TASSEL GUI for visualization of results prior to exporting the SNP calls as a spreadsheet [21].

SNPs were named according to scaffold and base pair position within the peach genome v1.0 build. SNP names contain the scaffold number, an underscore, and eight characters denoting the base position. Leading zeroes in the base position were retained, and a SNP at base 1,234,567 of scaffold 2 would therefore have been named ‘2_01234567’.

### Genetic map construction and QTL discovery

SNP data were converted to the ‘a, h, b’ codes with the female ‘Hakuho’ grandparent conferring the ‘a’ genotype. Because the F_1_ individual of this cross was deceased, SNPs in the F_2_ population were only used if the grandparental source of the alleles could unambiguously be assigned (i.e., one grandparent was homozygous at the SNP locus).

Genetic mapping of the F_2_ population was performed using JoinMap® 4.1 [22]. Highly similar markers (>0.95) were excluded from the data set to reduce calculation time. Where applicable, SSRs were preferentially retained from groups of SSR and SNP markers sharing similar segregation. Remaining markers were grouped using the ‘independence LOD’ function in JoinMap® with default settings. Marker groupings were manually verified by inspecting the grouped markers for agreement with known physical locations (SNPs) and known linkage group locations on the ‘T×E’ almond × peach reference map (SSRs) [13, 14, 17]. Marker order and distances were calculated using the Maximum Likelihood Mapping function with default settings. Segregation distortion of individual markers was calculated using the χ^2^ test in JoinMap. Markers with significant segregation distortion (*p*<0.01) were excluded if surrounding markers did not also show significant segregation distortion of a similar direction and magnitude.

QTLs were detected with MapQTL® 6 [23]. Genome-wise LOD significance thresholds were determined independently for each trait using the ‘Permutation Test’ function. The ‘Automatic Cofactor Selection’ tool was used iteratively to identify the strongest marker cofactors on each linkage group for each trait. Resulting cofactors were included in the search for QTLs that exceeded the LOD significance threshold. LOD curves were plotted with the MQM QTL detection algorithm.

## Results

### Sequencing and identification of SNPs

Illumina sequencing of 63 pooled, barcoded samples (1 sample per F2 individual, 3 samples per grandparent) generated approximately 1.5 million single-end 100 bp reads per sample. The final mean read number per genotype was 1,843,261 (+/- 1,092,631 SD) after merging genotypes with multiple barcodes and resequencing samples with low initial read numbers.

The TASSEL GBS pipeline initially identified 9,998 SNP loci distributed across all major scaffolds of the peach genome. Prior to the creation of a linkage map, data were filtered to remove SNPs with low read support. Within a genotype, data for individual SNPs were retained only if they possessed a minimum read depth of five and a minimum quality score of 98. After depth and quality filtering, SNPs for which data were missing in more than five genotypes were removed from further analysis. SNPs were also removed in grandparental genotype data were missing, if both grandparents shared a heterozygous genotype or if the minor allele frequency was less than 0.20. These filtering procedures resulted in 410 final SNP loci.

To estimate the effect of sequencing depth on SNP identification, we included the grandparental genotypes in triplicate in the sequencing pool (i.e. each grandparental genotype was tagged with three separate barcodes). This increased the combined sequencing depth of the grandparents threefold relative to the F_2_ individuals. Deeper sequencing of the grandparents (‘Hakuho’: 4.2 million reads, ‘UFGold’ 4.6 million reads) resulted in the identification of 4,833 final SNPs, approximately ten times more SNPs than were identified in the analysis of the F_2_ population. It should be noted that the decreased number of samples in this analysis (two grandparents vs. 57 F2s) results in fewer SNPs being discarded due to frequency of missing data across all individuals. The distribution of these grandparental SNPs on the physical map is shown in S1 Fig.

### Linkage mapping

**Fig 1:**
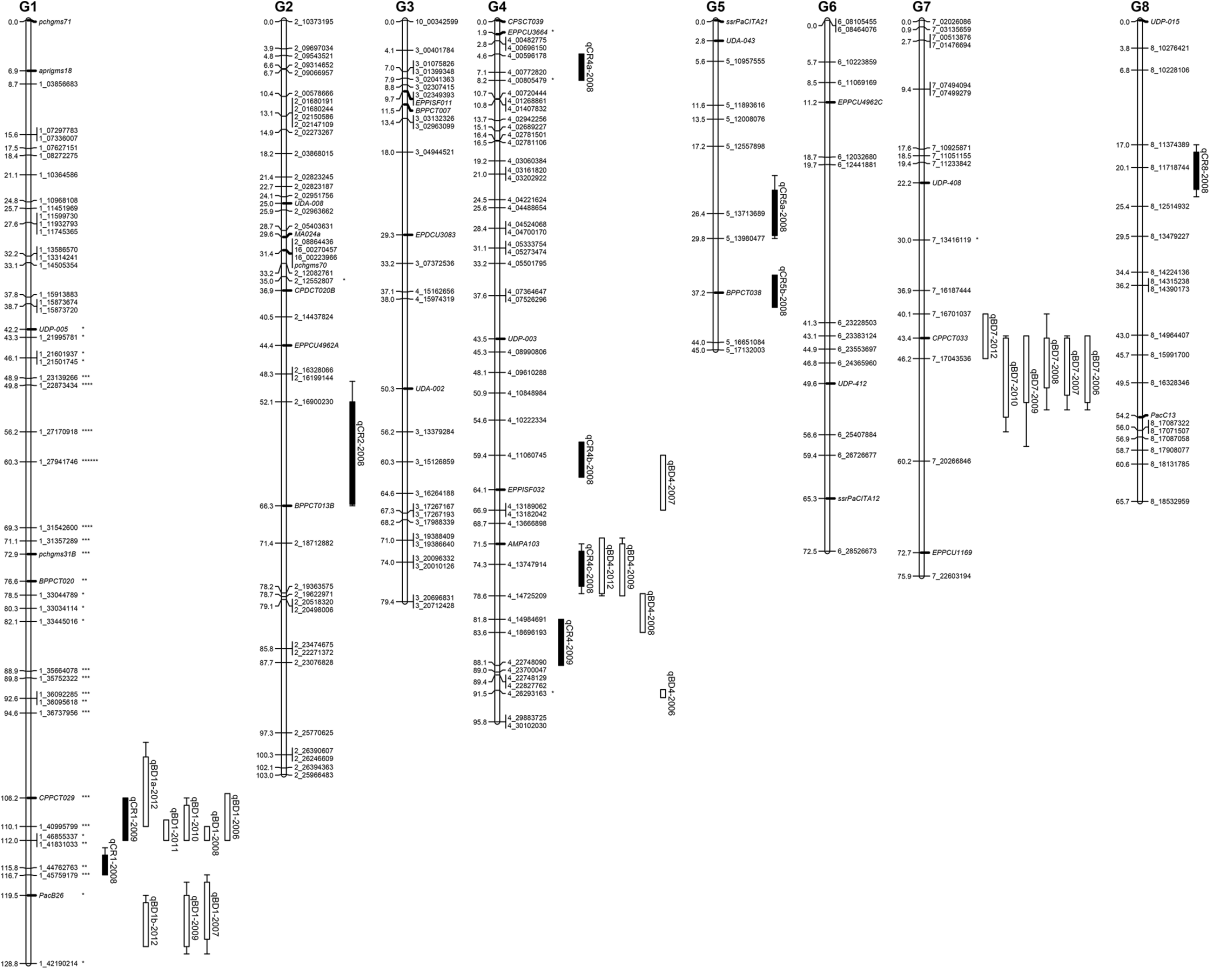
Linkage map of F_2_ mapping population segregating for chilling requirement (CR) and bloom date (BD). Linkage group numbering corresponds to the peach reference genetic map. Left side of bars are marker distances in cM. Labels on the right side of bars are the marker name. Markers with names followed by an asterix have significantly distorted genotypic ratios (*, *p*<0.05; **, *p*<0.01; ***, *p*<0.005; ****, *p*<0.001; *****, *p*<0.0005; ******, *p*<0.0001). Italicized labels are SSR markers, all other markers are SNPs and are named for the scaffold and bp position on the scaffold as found in the *Prunus persica* genome (v1.0). QTL intervals are shown to the left of each LG. Bars and whiskers on QTL represent the 1 LOD and 2 LOD confidence intervals from the QTL peak, respectively. Chilling requirement and bloom date QTLs are represented by solid and hatched bars, respectively. A QTL is named as qXXYa—ZZZZ, with ‘XX’ being the trait abbreviation, ‘Y’ the number of the linkage group, ‘a’ the letter to specify different QTLs for the same trait in one linkage group (G), and ‘ZZZZ’ the year in which the trait was phenotyped.

Of the initial 370 SSR markers screened, thirty-seven were identified as polymorphic in the F_2_ population. SSRs were combined with SNP markers identified from the TASSEL GBS pipeline and used to create a genetic linkage map. Following removal of SNPs with highly similar segregation or locally discontinuous segregation distortion, a linkage map was calculated using 201 SNPs and 33 SSR markers with an average intermarker distance of 2.85 cM. The resulting linkage map comprised eight linkage groups and a total map distance of 666.1 centiMorgans (Fig 1). One SNP from scaffold_10 and two SNPs from scaffold_4 of the peach genome v1.0 mapped to linkage group 3 (Fig 1). Two SNPs from scaffold_16 of the peach genome v1.0 mapped to linkage group 2 (Fig 1). Placement of these five SNPs is in agreement with the recent corrected assembly of the peach genome [17]. No SNPs were identified in the first several million bp of linkage groups 5, 6, and 8 (Fig 1).

Significant segregation distortion was observed for markers in the lower half of linkage group 1 (Fig 1). Segregation distortion in this region was caused by underrepresentation of genotypes homozygous for the allele derived from the high chill female grandparent ‘Hakuho’ and overrepresentation of genotypes homozygous for the allele derived from the low chill male grandparent ‘UFGold’.

### CR and BD phenotyping

**Fig 2:**
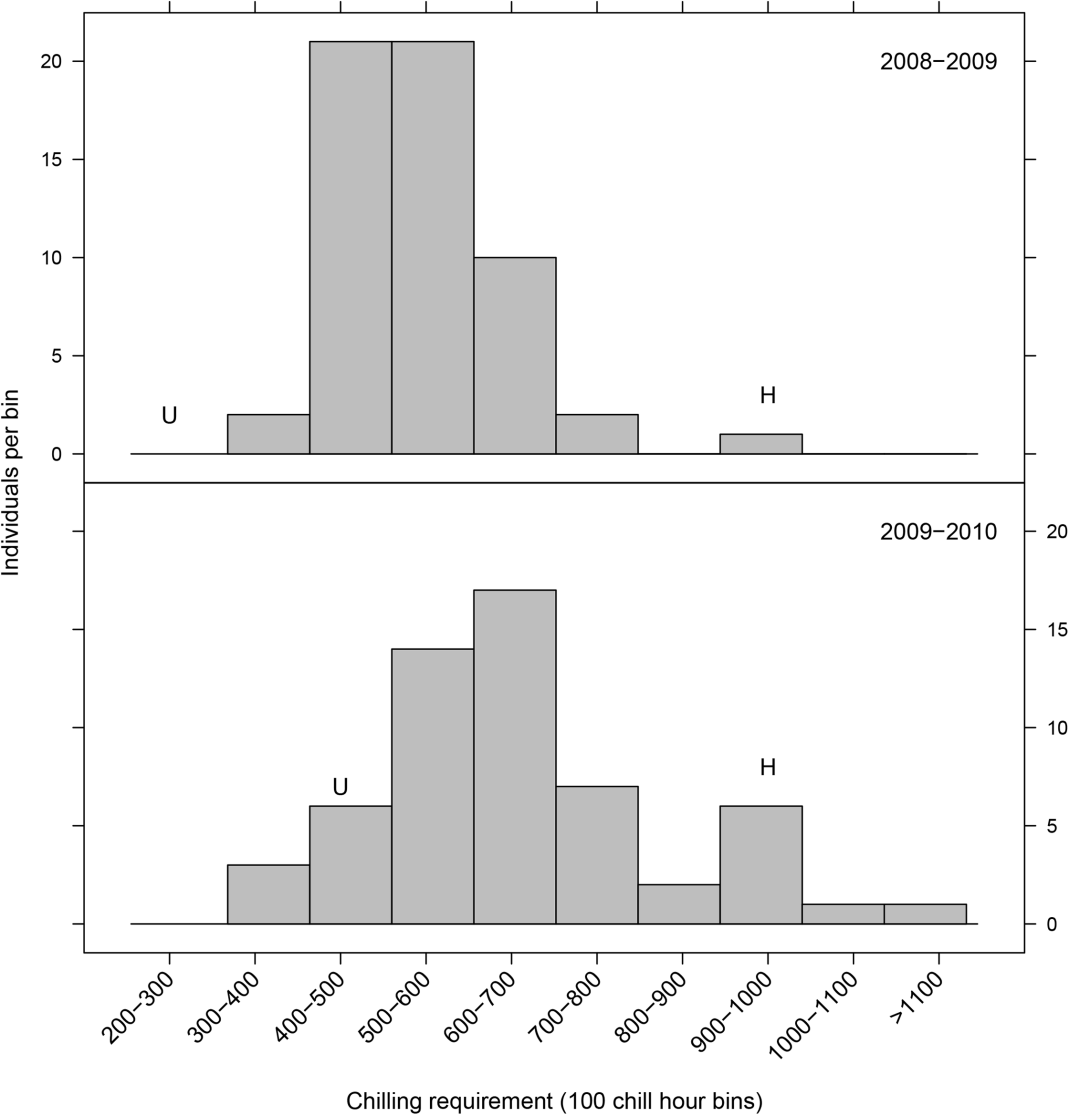
Histogram of chilling requirement (CR) phenotypes of F_2_ progeny in 2008/2009 and 2009/2010. Bins are 100 chill hour intervals. Letters indicate grandparental phenotypes (H, ‘Hakuho’; U, ‘UFGold’).

**Fig 3:**
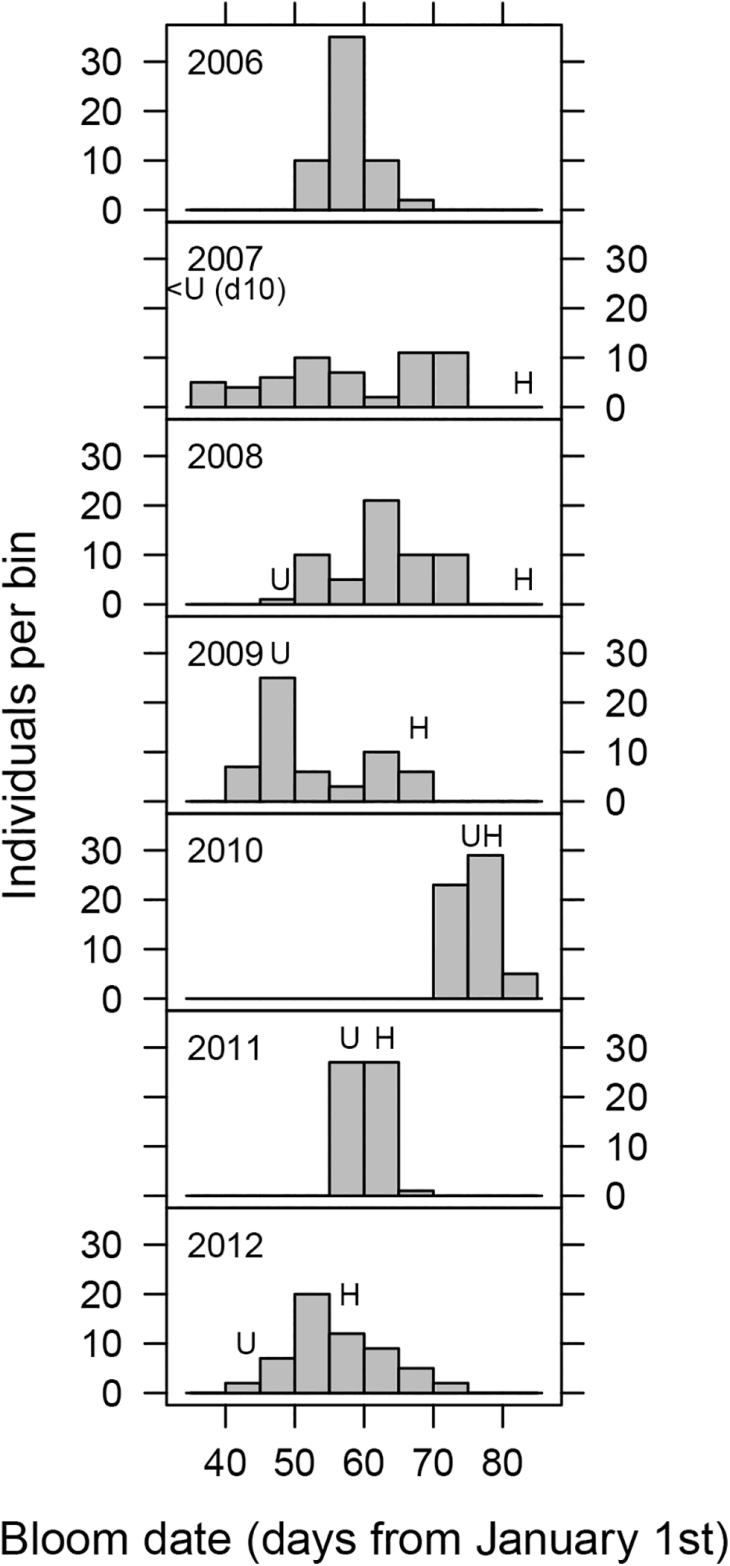
Histogram of bloom date phenotypes of F_2_ progeny in seven seasons of observations. Bloom date is measured from January 1^st^. Letters indicate grandparental phenotypes (H, ‘Hakuho’; U, ‘UFGold’).

F_2_ individuals segregated for both chilling requirement (CR) and bloom date (BD). CR ranged from as little as 300–400 chilling hours to >1100 chilling hours (Fig 2). BD varied strongly by year (Fig 3). The interval between the earliest and latest blooming F_2_ individuals varied with year from as little as 9 days (2010) to as long as 37 days (2007). Duration of the interval between earliest and latest bloom was reflective of year to year variation in chilling accumulation. Interruptions of chilling accumulation by warm periods promoted earlier bloom of low chill genotypes while consistent cold weather compressed bloom of all genotypes into a shorter interval (S2 and S3 Figs). Despite year to year variation in BD and earliest and latest BDs in the population, relative order of genotype BDs was highly correlated across years (Table 1). BD and CR phenotypes were also highly correlated (Table 1).

**Table 1:** Spearman’s rank order correlation coefficients for observed chilling requirement (CR) and bloom date (BD).

	CR2009	BD2006	BD2007	BD2008	BD2009	BD2010	BD2011	BD2012
CR2008	0.66	0.81	0.83	0.85	0.85	0.84	0.71	0.76
CR2009		0.66	0.59	0.62	0.59	0.60	0.59	0.55
BD2006			0.85	0.81	0.83	0.82	0.68	0.76
BD2007				0.92	0.90	0.90	0.82	0.87
BD2008					0.92	0.92	0.80	0.85
BD2009						0.89	0.79	0.82
BD2010							0.80	0.82
BD2011								0.67

Coefficients are all significant at the *p*<0.001 level

### QTL detection

Eight QTLs for CR were detected in 2008/2009 and two QTLs for CR were detected in the 2009/2010 (Fig 1, Table 2). Individual CR QTLs accounted for 4.0 to 27.8 percent of the phenotypic variation across years (Table 2). Nineteen QTLs were detected for BD across seven years of observations (Fig 1, Table 3). Individual BD QTLs accounted for 7.6 to 44.6 percent of phenotypic variation across years (Table 3). No CR or BD QTLs were identified on linkage groups 3 or 6 in any year.

**Table 2:** Chilling requirement QTL detected in 2008/2009 and 2009/2010.

Year	QTL	QTL Peak (cM)	Marker closest to peak	Peak LOD (Threshold LOD)	1 LOD interval (cM)	2 LOD interval (cM)	Flanking markers	Add.	*R* ^2^ (%)
**2008/2009**	qCR1-2008	115.8	1_44762763	12.78 (4.0)	115.0–116.7	112.0–116.7	1_41831033 / 1_46855337, 1_45759179	0.39	16.0
	qCR2-2008	61.0	2_16900230	7.72 (4.0)	53.5–66.1	49.3–66.3	2_16199144,BPPCT013B	-0.02	10.5
	qCR4a-2008	7.0	4_00772820	6.23 (4.0)	3.8–8.2	3.8–8.2	4_00772820, 4_00805479	-0.07	5.9
	qCR4b-2008	59.4	4_11060745	5.06 (4.0)	57.6–62.4	55.6–64.1	4_10222334,EPPISF032	-0.68	4.5
	qCR4c-2008	74.3	4_13747914	12.29 (4.0)	72.5–77.3	71.5–78.3	4_13666898, 4_14725209	1.09	14.9
	qCR5a-2008	26.4	5_13713689	6.00 (4.0)	23.2–29.4	21.2–29.8	5_12557898, 5_13980477	-0.004	5.7
	qCR5b-2008	37.2	BPPCT038	4.50 (4.0)	33.8–39.2	29.8–42.2	5_13980477, 5_16651084	0.28	4.0
	qCR8-2008	20.0	8_11718744	8.64 (4.0)	19.0–23.0	17.0–24.0	8_11374389, 8_12514932	-0.35	9.0
**2009/2010**	qCR1-2009	110.1	1_40995799	4.62 (3.6)	107.2–112.0	105.6–112.0	1_36737956, 1_41831033 / 1_46855337	0.16	24.8
	qCR4-2009	81.8	4_14984691	5.13 (3.6)	81.6–88.1	81.6–88.1	4_14725209, 4_22748090	1.11	27.8

A QTL is named as qXXYa—ZZZZ, with ‘XX’ being the trait abbreviation, ‘Y’ the number of the linkage group, ‘a’ the letter to specify different QTLs for the same trait in one linkage group (G), and ‘ZZZZ’ the year in which the trait was phenotyped.

**Table 3:** Bloom date QTL detected by year from 2006–2012. QTL are named as in Table 2.

Year	QTL	QTL Peak (cM)	Marker closest to peak	Peak LOD (Threshold LOD)	1 LOD interval (cM)	2 LOD interval (cM)	Flanking markers	Add.	*R* ^2^ (%)
**2006**	qBD1-2006	111.1	1_40995799	7.28 (3.6)	105.6–112.0	105.6–112.0	1_36737956, 1_46855337	1.15	26.4
	qBD4-2006	91.5	4_26293163	3.60 (3.6)	89.4–95.5	89.4–95.8	4_22748129, 4_29883725	1.15	11.2
	qBD7-2006	46.2	7_17043536	4.79 (3.6)	44.4–47.2	43.1–55.2	7_16701037, 7_20266846	1.38	15.6
**2007**	qBD1-2007	121.4	PacB26	14.17 (3.6)	117.7–125.4	116.7–127.4	1_45759179, 1_42190214	10.04	40.2
	qBD4-2007	66.1	4_13189062	4.43 (3.6)	58.6–66.9	58.6–66.9	4_10222334, 4_13189062	4.27	7.6
	qBD7-2007	46.2	7_17043536	14.45 (3.6)	43.4–51.2	43.1–53.2	7_16701037, 7_20266846	10.46	41.3
**2008**	qBD1-2008	111.1	1_40995799	17.42 (3.6)	110.1–112.0	110.1–112.0	1_36737956, 1_46855337	5.86	44.6
	qBD4-2008	81.8	4_14984691	6.23 (3.6)	78.3–83.6	78.3–83.6	4_13747914, 4_18696193	3.24	11.4
	qBD7-2008	46.2	7_17043536	8.90 (3.6)	43.4–50.2	43.1–53.2	7_16701037, 7_20266846	4.33	18.1
**2009**	qBD1-2009	121.4	PacB26	13.60 (3.6)	119.2–126.4	117.7–127.4	1_44762763, 1_42190214	6.77	44.4
	qBD4-2009	74.3	4_13747914	6.73 (3.6)	71.5–78.6	70.7–78.6	4_13666898, 4_14725209	4.58	17.0
	qBD7-2009	46.2	7–17043536	6.28 (3.6)	44.4–52.2	43.1–55.2	7_17701037, 7_20266846	4.53	15.5
**2010**	qBD1-2010	110.1	1_40995799	9.78 (3.6)	106.2–112.0	105.6–112.0	1_36737956, 1_41831033 / 1_46855337	1.69	35.9
	qBD7-2010	46.2	7–17043536	6.70 (3.6)	43.4–54.2	43.1–56.2	7_16701037, 7_20266846	1.43	21.1
**2011**	qBD1-2011	110.1	1_40995799	4.14 (4.1)	105.6–112.0	105.6–112.0	1_36737956, 1_41831033 / 1_46855337	0.98	29.4
**2012**	qBD1a-2012	104.6	CPPCT029	10.74 (3.6)	100.6–110.1	98.6–110.1	1_36737956, 1_40995799	4.88	36.5
	qBD1b-2012	123.4	PacB26	4.84 (3.6)	120.4–126.4	119.4–127.4	PacB26, 1_42190214	3.22	15.3
	qBD4-2012	75.3	4_13747914	7.77 (3.6)	70.7–78.3	70.7–78.6	4_13666898, 4_14725209	3.90	21.1
	qBD7-2012	43.4	CPPCT033	7.96 (3.6)	40.1–46.2	39.9–46.2	7_16187444, 7_17043536	3.71	20.9

## Discussion

### GBS is an effective genotyping method for peach

We successfully employed the GBS method to detect SNPs in a peach F_2_ mapping population. We employed the library preparation method as published with no modifications and were able to obtain a data set containing >1.5 million of *Ape*KI-associated reads per sample. TASSEL GBS pipeline 3.0 SNP calling appeared to be sensitive to low sequence depth in a species with greater heterozygosity than is typical of the cultivated cereals for which it was developed [20]. Nonetheless, after filtering all loci for read depth and genotype quality, mapping success with the remaining SNPs was greatly improved. Relatively stringent filtering of loci for minimum read depth, missing data and identifiable grandparental alleles reduced the number of SNP loci below that which has been typically reported in other species [24–26]. Relaxation of these filtering standards and the use of genotype imputation methods may increase the number of usable SNP loci obtained. The low background polymorphism of peach and the use of an F_2_ population may have also decreased the number of SNP loci relative to studies on unrelated individuals or F_1_ hybrids, which would likely possess greater allelic diversity and heterogeneity [24–26]. Finally, if increased SNP density were desired, reducing the number of individuals per sequencing lane would also increase the capture rate of SNPs in the population. We observed a nearly tenfold increase in detected SNPs when read depth was increased three-fold. GBS is, to some extent, a ‘tunable’ technique in which read depth can be adjusted to achieve a desired marker density.

Our ‘Hakuho’ × ‘UFGold’ population lacked detectable polymorphism in the distal ends of LGs 5 and 6 (Fig 1). These regions also lack observable polymorphisms between the grandparental genotypes (S1 Fig) and could represent either true monomorphic regions or regions with a very low density of *ApeK*I restriction sites. An *in silico ApeK*I digestion of the peach genome predicted that the greatest distance between two *ApeK*I sites in LG 5 and 6 would be approximately 75 and 25 kb, respectively. Since these two regions are several Mb in length, a complete absence of *ApeK*I sites is insufficient to explain the absence of SNPs. However, since the GBS procedure selects genomic fragments between 100 and 400 bp in length which are flanked by *ApeK*I sites, regions with more widely-spaced *ApeK*I sites would also appear to lack polymorphism.

Creation of merged or consensus linkage maps across independent populations is a valuable method for improving QTL localization. Creation of consensus maps relies on the existence of ‘anchor’ markers which have been genotyped across all populations being mapped. To assess the potential for an independent GBS experiment to find markers in common with SNPs from other research groups, we cross-referenced the physical locations of grandparental SNP loci against two published and publically-available peach SNP datasets. The first set of 6,557 SNPs (‘UCD’) was generated by GoldenGate^®^ technology to facilitate mapping of two F_2_ families segregating for fruit quality characters [3]. A second set of 9,000 SNPs (‘IGA_SNP’) was generated by re-sequencing of community-selected genotypes and curated for genome-wide representation [4]. Our pool of 4,833 GBS grandparental SNPs shared 33 SNP loci in common with the UCD dataset and 102 SNP loci in common with the IGA_SNP dataset (S2 File). The low number and uneven genomic distribution of shared SNP markers suggests that GBS-derived SNPs from a single population may have limited utility as anchoring markers for the integration of independent linkage maps (S2 File). This suggests a continued need for established anchoring markers currently used by the peach community to facilitate comparative mapping [13]. Conversion of novel SNPs discovered in one mapping project to cleaved amplified polymorphic sequence (CAPS) markers would also be a rapid and cost effective method to generate anchoring markers between maps of interest.

### Genetic control of CR and BD

We identified 29 QTLs for CR and BD in this F_2_ population across the two years of CR phenotyping and seven years of BD observation. Fewer QTLs were observed for CR than for BD. This discrepancy is consistent with the role of CR as a major, but not exclusive, determinant of BD. Additionally, more frequent BD observations allowed finer discrimination between genotypes than did the <10 bins of chill hour bins into which F_2_ genotypes were partitioned. 2010 and 2011 had notably fewer BD QTLs of all of the years (two and one, respectively). All other years had three or four QTL. The reduced number of QTL in these two years likely resulted from the relatively compact population variation in bloom in 2010 and 2011 (Fig 3). The temperatures in these two years was consistently cold through March and as a result most of the genotypes were saturated in their chilling accumulation. Most trees were therefore able to rapidly bloom with the occurrence of warm temperatures, reducing the differentiation between genotypes.

Across all years the three CR and BD QTLs with the greatest effect were those found on LG1, LG4, and LG7 (Tables 1 and 2). QTLs in the same regions have been identified in a number of peach mapping populations [8, 27, 28] and also appear to be conserved across other *Prunus* species [28–32].

Several QTLs regions overlap with locations of hypothesized gene candidates for the genetic control of CR and BD. One group of strong CR and BD QTLs at the end of LG1 corresponds to the genomic location of the peach *DAM* gene cluster responsible for the *evg* mutation [33, 34]. In three years, QTLs for BD were detected within 10 cM of the DAM gene cluster, suggesting that this genomic region may also contain other candidates (Fig 1). The CR QTL located on LG2 (qCR2-2008) spans a region of the genome that contains *PpeMADS22*, a peach homolog of *ParSOC1*, that has been implicated in control of CR for vegetative bud break in *P*. *armeniaca* [32, 35, 36].

Our ‘Hakuho’ × ‘UFGold’ population displayed a strong segregation distortion at the bottom of LG1 as previously observed in a study of the unrelated ‘Contender’ × ‘FLA92-2C’ population [8]. Identification of similar segregation distortion in an unrelated cross is consistent with a linkage between the low chill grandparent derived alleles and a locus that distorts segregation in this region. However, the seeds of both of these populations (‘Hakuho’ × ‘UFGold’ and ‘Contender’ × ‘FLA92-2C’) were harvested, stratified and germinated in the same season and location. This allows the possibility that environmental selection occurred at an early developmental stage, biasing the resulting populations in a similar direction.

## Conclusions

GBS is a rapid and cost-competitive method for genotyping in peach. In our example, 96 individuals were multiplexed on a single lane of an Illumina HiSeq® 2000 machine to produce a dataset of several hundred SNPs for use in linkage mapping. Assuming a conservatively high sequencing cost of $2000 per lane, this is approximately $21 per sample excluding the initial expense of the adaptor oligonucleotides, which can be amortized over >200 sequencing runs. Although this cost is still prohibitive for large scale breeding programs that screen thousands of individuals annually, it is competitive for genotyping populations in trait mapping studies. As sequencing costs continue to decline, the cost competitiveness of this method will only improve.

## Supporting Information

S1 Fig Physical positions of SNP markers polymorphic between the ‘Hakuho’ and ‘UFGold’ grandparental genotypes. Physical map of peach chromosomes (vertical bars) with horizontal marks indicating position of SNPs between grandparental genotypes.
(TIF)

S2 Fig Cumulative chill hours below 7 degrees C in the seven winters of bloom date observation. (TIF)

S3 Fig Cumulative chill hours between 0–7 degrees C in the seven winters of bloom date observation. (TIF)

S1 File Scripts for extracting barcode sequences from raw fastq files. (PDF)

S2 File List of peach SNPs in common between this study, IGA_SNP, and UCD datasets. (XLSX)
