# Supplementary figures and images for: Genotyping by Sequencing for SNP-Based Linkage Map Construction and QTL Analysis of Chilling Requirement and Bloom Date in Peach [Prunus persica (L.) Batsch]
Source: PLoS One. 2015 Oct 2;10(10):e0139406. doi: 10.1371/journal.pone.0139406 (PMC4592218; doi:10.1371/journal.pone.0139406)

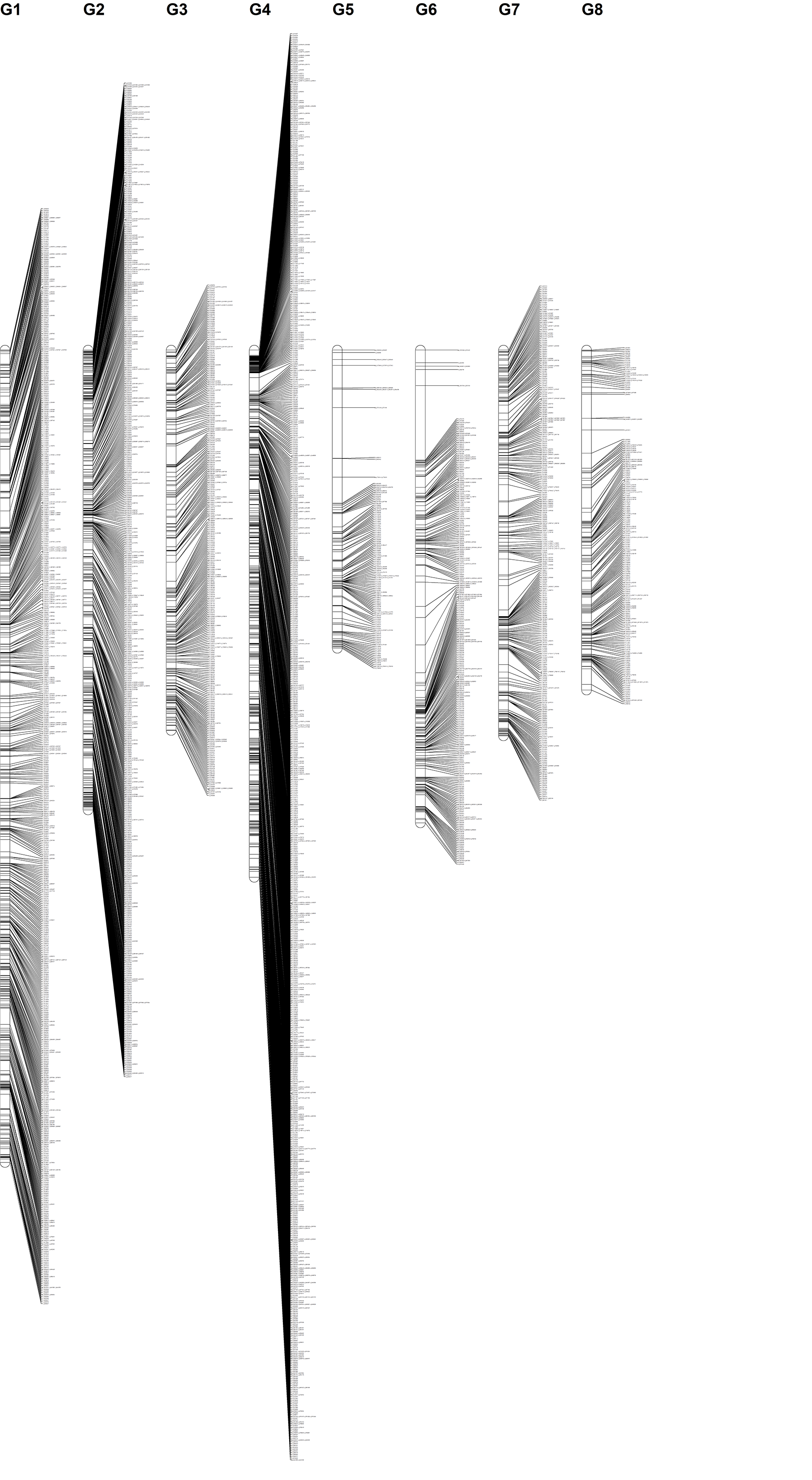

Supplement: S1 Fig — Physical map of peach chromosomes (vertical bars) with horizontal marks indicating position of SNPs between grandparental genotypes. (TIF) [file pone.0139406.s001.tif]

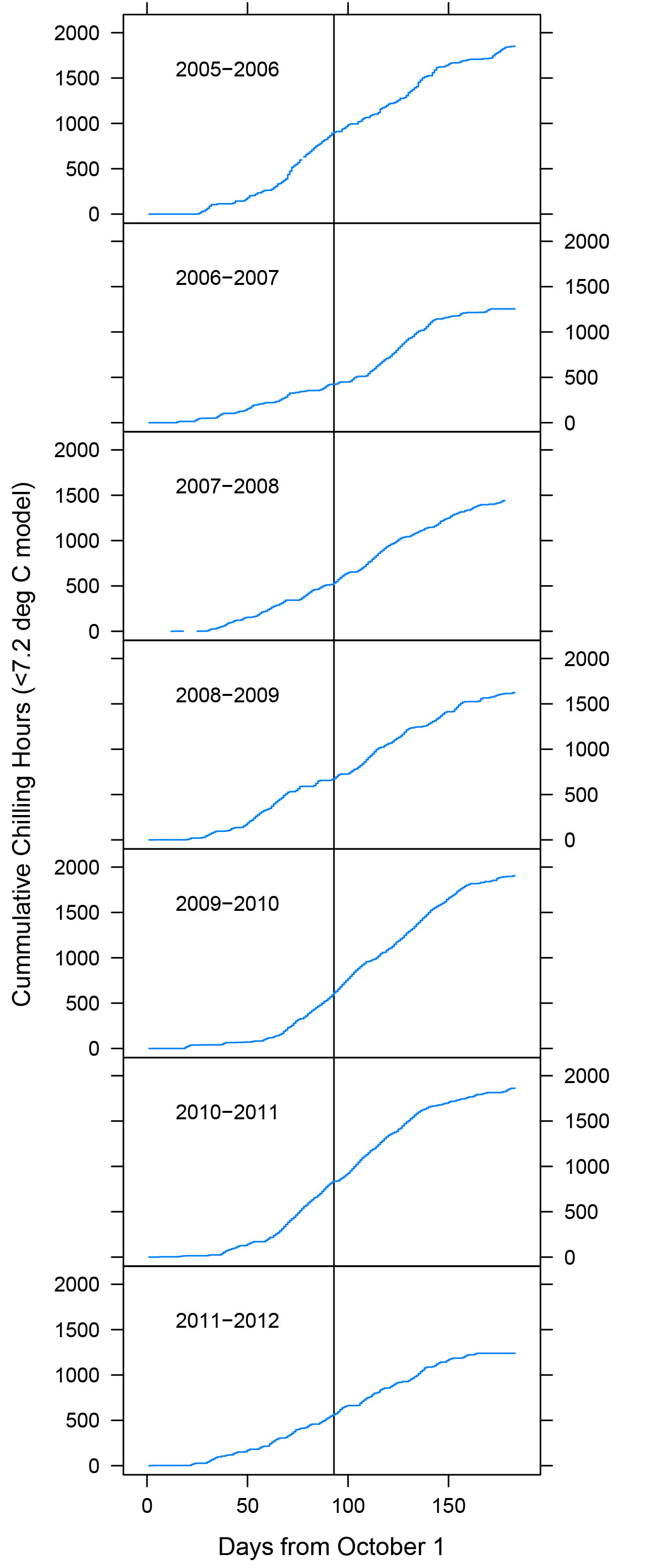

Supplement: S2 Fig — (TIF) [file pone.0139406.s002.tif]

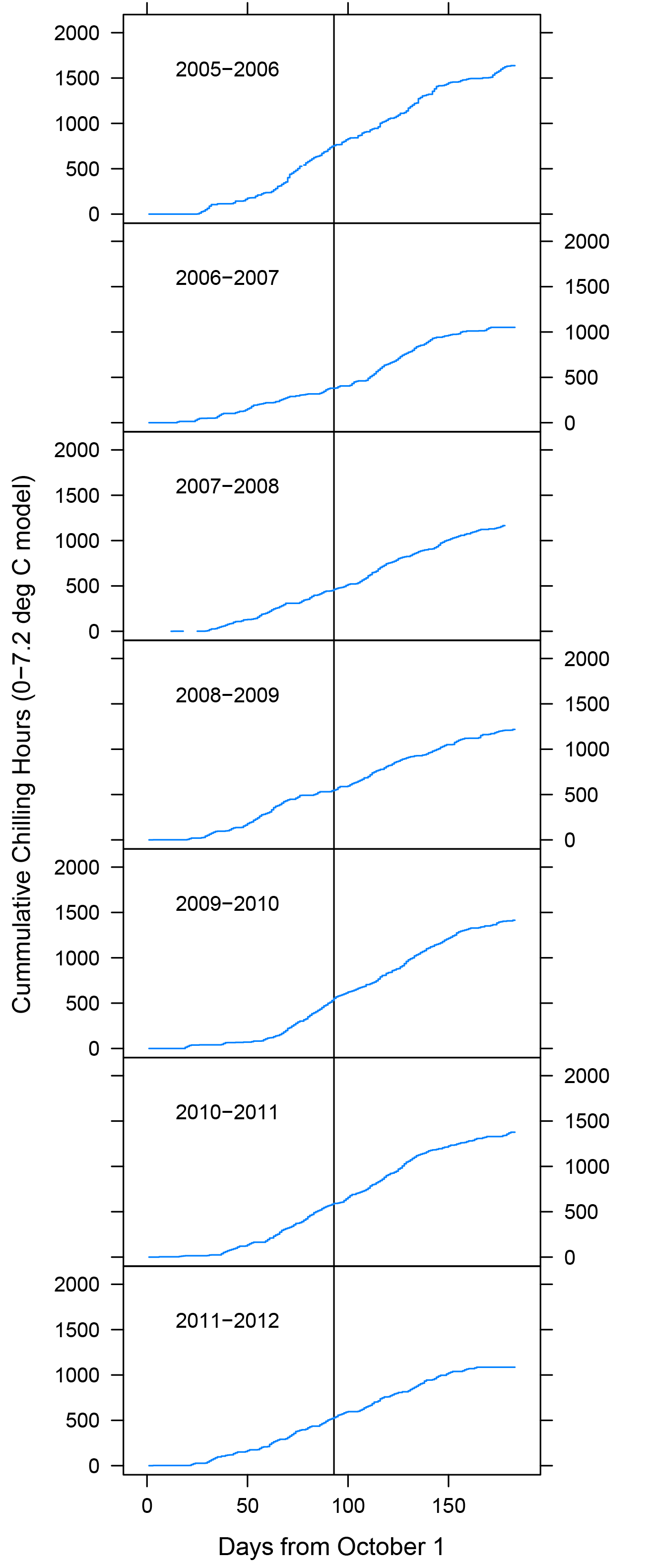

Supplement: S3 Fig — (TIF) [file pone.0139406.s003.tif]
