# Supplementary material for: Genotyping by Sequencing for SNP-Based Linkage Map Construction and QTL Analysis of Chilling Requirement and Bloom Date in Peach [Prunus persica (L.) Batsch]
Source: PLoS One. 2015 Oct 2;10(10):e0139406. doi: 10.1371/journal.pone.0139406 (PMC4592218; doi:10.1371/journal.pone.0139406)
Supplement: S1 File — (PDF) [file pone.0139406.s004.pdf]

****
Note: this process assumes you've got Homer (http://biowhat.ucsd.edu/homer/) installed and Homer's PATH information in your .bashrc
****

Working from the directory that contains your read file (file.fastq):

```
$ cat file.fastq | perl -e '$i=0;while(<>){if(/^\@/&&$i==0){s/^\@/\>/;print;}elsif($i==1){print;$i=-3}$i++;}' > file.fasta
```

```
$ homerTools trim -3 CAGC file.fasta
```

```
$ homerTools trim -3 CTGC file.fasta.trimmed
```

```
$ awk '0 == NR % 2'  file.fasta.trimmed.trimmed  > barcodes.txt
#removes fasta identifiers and keeps only the barcode seqs
```

```
$ sed '/.\{9\}/d' barcodes.txt >barcodes_final.txt
#this drops "barcodes" longer than 8 characters - which are likely artifacts
```

```
$ cat barcodes_final.txt |sort |uniq -c > barcode_counts
```
